# Classical determinants of coronary artery disease as predictors of complexity of coronary lesions, assessed with the SYNTAX score

**DOI:** 10.1007/s12471-017-1005-0

**Published:** 2017-06-07

**Authors:** J. M. Montero-Cabezas, I. Karalis, R. Wolterbeek, A. O. Kraaijeveld, I. E. Hoefer, G. Pasterkamp, N. H. Pijls, P. A. Doevendans, J. Walterberger, J. Kuiper, A. J. van Zonneveld, J. W. Jukema

**Affiliations:** 1 Department of Cardiology C5-P, Leiden University Medical Center, Leiden, The Netherlands; 2 Department of Medical Statistics, Leiden University Medical Center, Leiden, The Netherlands; 3 Department of Cardiology, University Medical Center Utrecht, Utrecht, The Netherlands; 4 Laboratory of Experimental Cardiology, University Medical Center Utrecht, Utrecht, The Netherlands; 5 Department of Cardiology, Catharina Hospital, Eindhoven, The Netherlands; 6 Department of Cardiology, Maastricht University Medical Center, Maastrischt, The Netherlands; 7 Department of Cardiovascular Medicine, University Hospital Münster, Münster, Germany; 8 Department of Biopharmaceutics, Leiden University Medical Center, Leiden, The Netherlands; 9 Department of Internal Medicine (Nephrology), Leiden University Medical Center, Leiden, The Netherlands; 10 Einthoven Laboratory for Experimental Vascular Medicine, Leiden University Medical Center, Leiden, The Netherlands

**Keywords:** Coronary artery disease, Coronary angiography, Risk assessment, Risk factors

## Abstract

**Background:**

We need new biomarkers that can predict cardiovascular disease to improve both diagnosis and therapeutic strategies. The CIRCULATING CELLS study was designed to study the role of several cellular mediators of atherosclerosis as biomarkers of coronary artery disease (CAD). An objective and reproducible method for the quantification of CAD extension is required to establish relationships with these potential biomarkers. We sought to analyse the correlation of the SYNTAX score with known CAD risk factors to test it as a valid marker of CAD extension.

**Methods and results:**

A subgroup of 279 patients (67.4% males) were included in our analysis. Main exclusion criteria were a history of previous percutaneous coronary intervention or surgical revascularisation that prevent an accurate assessment of the SS. Diabetes mellitus, smoking, renal insufficiency, body mass index and a history of CAD and myocardial infarction were all positively and strongly associated with a higher SYNTAX score after adjustment for the non-modifiable biological factors (age and sex). In the multivariate model, age and male sex, along with smoking and renal insufficiency, remain statistical significantly associated with the SYNTAX score.

**Conclusion:**

In a selected cohort of revascularisation-naive patients with CAD undergoing coronary angiography, non-modifiable cardiovascular risk factors such as advanced age, male sex, as well as smoking and renal failure were independently associated with CAD complexity assessed by the SYNTAX score. The SYNTAX score may be a valid marker of CAD extension to establish relationships with potential novel biomarkers of coronary atherosclerosis.

## Introduction

The search for new biomarkers that predict cardiovascular disease has become a priority in the need to improve early diagnosis and establish individual treatment strategies. The CIRCULATING CELLS study was designed to study a broad spectrum of features associated with circulating hematopoietic cellular subsets as biomarkers of coronary artery disease (CAD) and atherosclerosis [1].

In order to analyse potential relationships of novel markers with the extension of CAD, reproducible and objective methods for coronary atherosclerosis quantification are needed. The Synergy between Percutaneous Coronary Intervention with Taxus and Cardiac Surgery (SYNTAX) score (SS) has emerged as a tool to objectively quantify CAD complexity from an anatomical point of view, offering prognostic information and guidance concerning the appropriate coronary revascularisation method [2]. Since its introduction, SS has also been used as a method to provide objective and reproducible anatomical information about the extent of CAD [3].

Classical determinants of cardiovascular disease such as age, male gender, hypertension, dyslipidaemia, diabetes and smoking have been linked to more extensive forms of CAD [4]. A more complex disease pattern, reflected by a higher SS, is therefore expected in patients with a higher risk profile. The evidence associating SS with a higher risk profile, is however still scarce [5].

We sought to analyse the correlation of SS, as a marker of CAD extension, with traditional and other known risk factors related with the development of coronary atherosclerosis in participants of the CIRCULATING CELLS study [1].

## Methods

### Study cohort

The cohort consists of 279 subjects, representing a subgroup of the CIRCULATING CELLS study population. The CIRCULATING CELLS study was a multicentre, prospective study conducted from March 2009 to September 2011 in four medical centres in the Netherlands [1]. The total number of patients enrolled was 714. For the needs of our analysis, patients with previous history of coronary intervention (percutaneous coronary intervention, or coronary artery bypass grafting) were not included.

According to the study protocol, described in detail elsewhere [1], patients admitted with chest pain and a provisional diagnosis of stable angina, unstable angina or non-ST-segment elevation myocardial infarction (NSTEMI) were considered eligible for inclusion. Patients presenting with ST-segment elevation myocardial infarction were excluded. All patients underwent a diagnostic coronary angiography. Blood samples were collected at inclusion and stored for further analysis. We entered the obtained data into a centralised database.

The study was approved by the ethics committee at each participating centre and conforms to the declaration of Helsinki. All patients provided written informed consent at inclusion.

### Assessment of cardiovascular risk determinants

The CIRCULATING CELLS database was used to obtain information about the prevalence of cardiovascular risk determinants. Age, sex, hypertension, diabetes, dyslipidaemia, smoking habits, family history of premature coronary artery disease, renal insufficiency, prior history of myocardial infarction and prior peripheral and cerebrovascular disease data were collected. Body mass index (BMI) was calculated and included in the analysis.

Hypertension was considered present when systolic blood pressure was ≥140 mm Hg and/or diastolic blood pressure ≥90 mm Hg or in the event of chronic use of blood pressure-lowering agents. Diabetes was considered present if the subject was treated with insulin or oral hypoglycaemic drugs or if fasting serum glucose was ≥7.0 mmol/l or serum glucose ≥11.1 mmol/l at admission. Dyslipidaemia was defined as total cholesterol >5.0 mmol/l, low-density lipoprotein (LDL) cholesterol >3.2 mmol/l or the use of lipid-lowering drugs. Patients who had been smoking regularly within 12 months prior to the inclusion were considered smokers.

Renal insufficiency was considered present if previously reported or serum creatinine value measured at inclusion >150 μmol/l. Cerebrovascular disease was considered present if previous history of transient ischaemic attack, cerebral infarction, cerebral ischaemia or amaurosis fugax had been reported. Peripheral artery disease was defined as a symptomatic and documented obstruction of distal arteries of the leg or interventions, or history of abdominal or infrarenal aortic aneurysm.

### SS measurements

All coronary angiograms obtained at inclusion were evaluated at the Leiden University Medical Centre by two experienced interventional cardiologists. SS was calculated with the use of the online calculator and following the definitions of the SYNTAX study [2] (www.syntaxscore.com). In cases of disagreement, the opinion of a third analyst was obtained and the final decision was made by consensus.

### Statistical analysis

Continuous variables are expressed as mean ± standard deviation (SD) and categorical variables are expressed as percentages. Using the general linear model, univariate analysis was performed to identify the determinants that would further be included in a multivariate regression model. Variables with a *p*-value of <0.15 in the univariate analysis were considered eligible. An additional univariate analysis was performed, adjusting the determinants for age and sex which are considered non-modifiable biological factors. In the multivariate model, a *p*-value of <0.05 was considered significant. Statistical analysis was performed with SPSS (SPSS v.22, Chicago, IL).

## Results

Two hundred and seventy-nine patients were included (mean age 60.9 ± 10.4 years). The prevalence of classical risk determinants for CAD and baseline characteristics are presented in Table 1.

**Table 1:** Baseline characteristics

	CIRCULATION CELLS study population, treatment-naive patients (*N* = 279)
*Syntax score (median, IQR)*	11 (4, 18)
*Male (n, %)*	188 (67.4%)
*Age in years (mean, SD)*	60.9 ± 10.4
*Race (n, %)*	
Caucasian	266 (95.3%)
Hindu	1 (0.4%)
Asian	9 (3.2%)
Other	3 (1.1%)
*Hypertension (n, %)*	162 (58.1%)
*Diabetes mellitus (n, %)*	54 (19.4%)
*Dyslipidaemia (n, %)*	164 (58.8%)
*Smoking habits (n, %)*	
Active Smokers	59 (21.1%)
Ex-smokers	29 (10.4%)
Never smokers	190 (68.1%)
*Familiar history premature CVD (n, %)*	133 (47.7%)
*BMI (mean, SD) kg/m* ^2^	27.2 ± 4.1
*PVD (n, %)*	29 (10.4%)
*Renal insufficiency (n, %)*	3 (1.1%)
*CVA/TIA (n, %)*	20 (7.2%)
*Previous MI (n, %)*	40 (14.3%)
*LVEF (n, %)*	
>50%	99 (35.5%)
30–50%	17 (6.1%)
<30%	2 (0.7%)
Unknown/missing data	182 (65.3%)
*Salicylates*	216 (77%)
*Clopidogrel*	109 (39%)
*ACE inhibitors*	77 (28%)
*ARB*	45 (16%)
*Statines*	198 (71%)
*Beta-adrenergic blocking agent*	187 (67%)
*Calcium antagonists*	56 (20%)
*Nitrates*	83 (30%)

*BMI* body mass index, *CVD* cardiovascular disease, *PVD* peripheral vascular disease, *CVA/TIA* cerebrovascular accident/transient ischaemic attack, *MI* myocardial infarction, *ACE* angiotensin converting enzyme, *ARB* angiotensin receptor blockers, *LVEF* left ventricular ejection fraction, *IQR* interquartile range, *SD* standard deviation

**Fig. 1:**
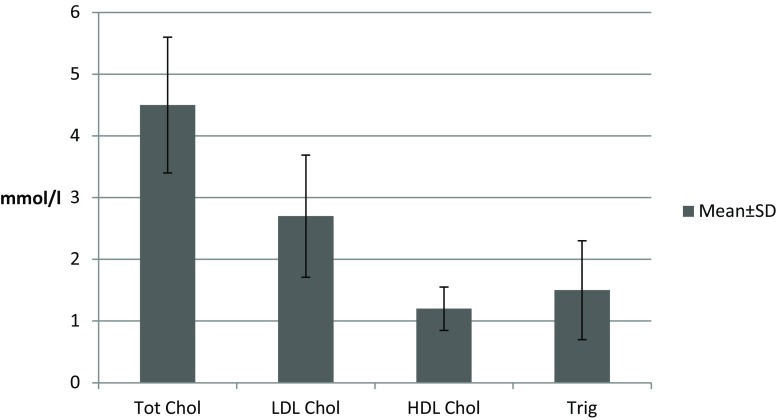
Lipid profile of included patients at inclusion. (*Tot chol* total cholesterol, *LDL chol* low-density lipoprotein cholesterol, *HDL chol* high-density lipoprotein cholesterol, *trig* triglycerides, *SD* standard deviation)

The clinical setting of presentation and the calculated SS per group are provided in Table 2. The majority of the patients presented with stable angina (209 patients, 74.9%), whereas 15 (5.9%) presented with unstable angina and 36 (12.9%) with NSTEMI; there were 19 patients included in the study where, despite the complaints, no significant CAD was identified (and therefore the SS was given as zero). Despite the fact that this study included coronary intervention-naive patients, the vast majority was already using antiplatelet agents and statins at inclusion. The latter is reflected in the lipid profile and the relatively low LDL cholesterol values presented in Fig. 1.

**Table 2:** Clinical syndrome and syntax score based on presentation

Clinical Syndrome	*N* (%)	Syntax score (median, IQR)
Stable angina	209 (74.9%)	11 (5, 19)
Unstable angina	15 (5.4%)	15 (7, 18)
NSTEMI	36 (12.9%)	16 (9, 24.5)
Atypical thoracic pain/non-significant CAD	19 (6.8%)	0 (0, 0)

*NSTEMI* non-ST-segment elevation myocardial infarction, *CAD* coronary artery disease, *IQR* interquartile range

**Fig. 2:**
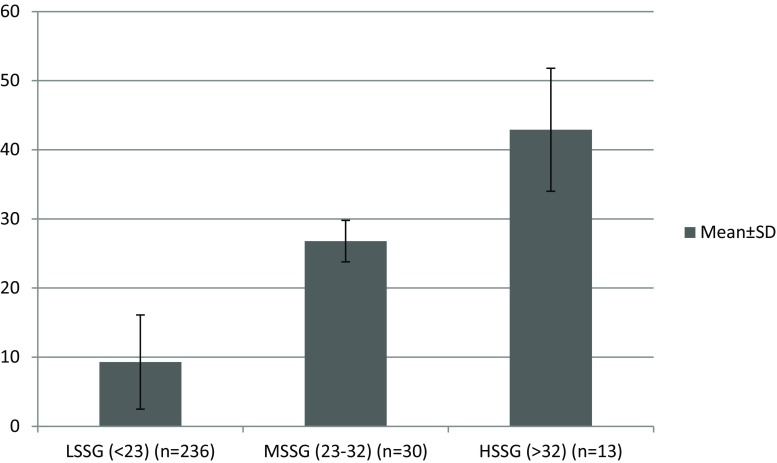
Distribution of Syntax scores in the included population. (*LSSG* low syntax score group, *MSSG* medium syntax score group, *HSSG* high syntax score group, *SD* standard deviation)

The calculated SS of the total population had a median value of 11 (IQR: 4, 18). The majority of the patients (*n* = 236) had a calculated low SS (LSS, <23), 30 patients had a medium SS (MSS, 23–32) while 13 were found with a high SS (HSS, >32). The median values and interquartile range (IQR) of each group are presented in Fig. 2.

The results of the univariate and multivariate analysis are presented in Tables 3 and 4. Age was clearly associated with a higher SS, while male sex did not reach statistical significance. After adjustment for the non-modifiable biological factors (age and sex), diabetes mellitus, smoking, renal insufficiency, body mass index and a history of CVD and myocardial infarction are all positively and strongly associated with a higher SS. In the multivariate analysis following a general linear model, age and male sex were identified as significant independent risk factors (age: regression coefficient 0.185, *p* = 0.007, male: 3.488, *p* = 0.012); the association of other determinants with SS is eliminated except for renal insufficiency and smoking (renal failure, regression coefficient: 13.737, *p* = 0.029, smoking regression coefficient: 3.889, *p* = 0.009).

**Table 3:** Univariate and sex/age corrected analysis of risk factors, as determinants of SYNTAX score

	Univariate regression coefficient (beta’s)	95% CI	*p*-value	Regression coefficient adjusted for age and sex	95% CI	*p*-value
*Age*	0.140	0.019, 0.261	*0.024*	–	–	–
*Male sex*	2.569	−0.148, 5.287	0.064	–	–	–
*Diabetes mellitus*	3.745	0.530, 6.960	*0.023*	3.285	0.083, 6.487	*0.044*
*Hypertension*	2.521	−0.060, 5.102	0.056	2.380	−0.233, 4.992	0.074
*Dyslipidaemia*	1.877	−0.719, 4.4.72	0.156	2.049	−0.517, 4.615	0.117
*Renal insufficiency*	17.467	5.209, 29.726	*0.005*	16.664	4.398, 28.930	*0.008*
*History of CVA/TIA*	4.341	−0.603, 9.284	0.085	4.356	−0.544, 9.256	0.081
*Previous MI*	4.558	0.940, 8.177	*0.014*	4.344	0.768, 7.920	*0.017*
*Smoking* ^*a*^	2.144	−0.610, 4.898	0.127	3.884	1.010, 6.759	*0.008*
*BMI*	0.394	0.076, 0.712	*0.015*	0.422	0.108, 0.736	*0.009*

*CVA/TIA* cerebrovascular accident/transient ischemic attack, *MI* myocardial infarction, *BMI* body mass index, *CI* confidence interval

^a^Ever smokers/current smokers versus never smokers

**Table 4:** Multivariate model analysis of risk factors as determinants of SYNTAX score

	Regression coefficient (betas)	95% CI	*p*-value
*Age*	0.186	0.053, 0.320	*0.006*
*Male sex*	3.454	0.754, 6.155	*0.012*
*Diabetes mellitus*	1.902	−1.529, 5.332	0.276
*Hypertension*	1.280	−1.463, 4.022	0.359
*Dysplipidaemia*	1.249	−1.437, 3.935	0.361
*Renal insufficiency*	13.737	1.397, 26.077	*0.029*
*History of CVA/TIA*	4.022	−1.213, 9.257	0.132
*Previous MI*	2.921	−0.709, 6.551	0.114
*Smoking* ^*a*^	3.889	0.984, 6.794	*0.009*
*BMI*	0.223	−0.111, 0.557	0.189

*CVA/TIA* cerebrovascular accident/transient ischemic attack, *MI* myocardial infarction, *BMI* body mass index, *CI* confidence interval

^a^Ever smokers/current smokers versus never smokers

## Discussion

Our analysis examines the relationship of SS with traditional cardiovascular risk factors in a selected population of patients undergoing coronary angiography from the CIRCULATING CELLS study. We demonstrate a positive correlation with increased age, as well as the presence of diabetes mellitus, smoking habit and obesity. A positive correlation is also demonstrated with renal insufficiency and, as expected, with previously established CAD (in the form of previous myocardial infarction). In the multivariate analysis model, age, male sex, history of smoking and renal insufficiency remained as predictors of an increased SS.

An accessible and reproducible method to evaluate the angiographic extension of CAD is mandatory for further analysis associating potential biomarkers and coronary atherosclerosis severity. SS has become an indispensable tool to evaluate CAD complexity and to guide the revascularisation approach election [2, 6]. Recently, it has been demonstrated that the SYNTAX score II guides the revascularisation strategy choice better by combining SS with a number of clinical variables [7]. For the purpose of our study, SS was chosen due to its strict anatomical-based design to assess CAD complexity. Current coronary revascularisation guidelines advocate the use of SS to determine the revascularisation modality [8, 9], despite its limitations [10] and despite the fact that criticism is being raised whether the conclusions of the SYNTAX trial still apply in current clinical practice with the use of 2nd and 3rd generation drug-eluting stents [11]. SS has also been used as a surrogate marker of CAD extent in studies which sought to establish correlations of several clinical and biochemical variables with coronary atherosclerosis [5, 12, 13]. However, SS in this context has not been properly validated.

The role of age, male gender, smoking, diabetes mellitus and obesity as determinants of CAD has been discussed extensively since the publication of the Framingham Heart study and a series of landmark studies indicating a causal relationship with atherosclerosis [14, 15]. Our study suggests a positive association of these parameters with the complexity of CAD, as expressed through SS. Aging is associated with progressive endothelial dysfunction, occurring earlier in males, presumably due to the protective role of oestrogens in pre-menopausal women [16]. Smoking affects all phases of atherosclerosis, from endothelial dysfunction to acute clinical events [17]. In diabetes hyperglycaemia, insulin resistance and free fatty acid release have been shown to lead to increased oxidative stress and therefore accelerate atherosclerosis [18]. Diabetes was not however a predictor of SS in the multivariate analysis in our study. Although this could be explained by the relatively small sample size, it should be noticed that only coronary lesions located in vessels with diameters >1.5 mm qualify for SS calculation. Therefore, CAD extension in diabetic patients might be underestimated with this method.

The role of BMI as predictor of CAD is controversial. In a study evaluating 13,874 patients referred for computed tomographic angiography, an increased BMI was associated to a higher prevalence, extension and severity of CAD and increased risk of myocardial infarction [19]. BMI was also a predictor of CAD but not of its severity in another similar study including 1706 patients [20]. On the other hand, an inverse relationship of obesity with death in patients with known cardiovascular disease is well known and described as the ‘obesity paradox’ [21, 22]. These findings may be related to several factors, as the inability of BMI to discriminate between excessive amounts of body fat and increments of lean mass or the introduction of more aggressive secondary prevention strategies in patients with high BMI. Based on our results, BMI might not be reliable as a clinical marker of complex CAD. Other parameters focused on body fat distribution, such as the presence of central obesity, waist circumference or waist-to-hip ratio, have been related to higher rates of myocardial infarction or even mortality. Further analysis is required to determine their association with SS.

Although the number of patients with renal insufficiency in our study is limited and prevents us from drawing definitive conclusions, our findings are in accordance with a previous study comprising 2262 patient who underwent coronary angiography, where kidney function was found to be an independent predictor of SS [23].

The lack of correlation with other well-known vascular risk factors, such as dyslipidaemia or hypertension, most likely reflects the impact of prevention strategies in this population. A high number of patients undergoing CAG are treated with statins in current practice. Statins reduce the concentration of circulating LDL cholesterol and other apo-B-containing lipoproteins, reduce moderately elevated triglycerides levels and elevate HDL cholesterol levels up to 5–10%. Besides, they have been proven to reduce plaque burden and induce plaque stabilisation [24]. Angiotensin-II receptor blocking agents, broadly used in patients at high vascular risk, are linked to lower rate of coronary atheroma progression [25]. Statins were used by 71% of the individuals of our cohort, angiotensin converting enzyme inhibitors by 28% and angiotensin receptor blockers by 16% at inclusion. Therefore, the association of potentially modifiable risk factors, such as hypertension and dyslipidaemia, and extension or complexity of CAD may have become spurious.

As mentioned above, SS has been used as a surrogate marker of CAD and has been compared with several biological variables potentially implicated in the development of coronary atherosclerosis as fasting blood glucose, monocyte subtypes, red cell distribution width or bilirubin levels [26–28]. We believe that the demonstrated association of ‘non-modifiable’ risk factors with the complexity of CAD legitimates the use of SS in this scenario.

## Limitations

Although this is a prospective study, the study cohort was selected among patients included in the CIRCULATING CELLS trial following specific inclusion criteria. Thus, patients with STEMI, previous bypass or previous coronary interventions were excluded. Since these patients theoretically have more extended CAD, their exclusion may condition a selection bias. The relatively small sample size and the limited number of the analysed baseline conditions might have an influence in the observed results.

SS limitations should be addressed. Only coronary stenosis ≥50% in vessels with a diameter ≥1.5 mm qualify for scoring. Lumen reductions below 50% are therefore excluded. Hence, a patient with a focal 70% stenosis in the proximal circumflex artery with no other lesions will have a higher SS than a patient with 40% lesions in multiple segments. This illustrates that higher SS values do not necessarily imply more extended atherosclerosis. SS calculation relies exclusively on a visual evaluation of the coronary angiography, implying potential misinterpretation and inter-observer variability [10, 29].

## Conclusion

In a selected cohort of revascularisation-naive patients with CAD undergoing coronary angiography, non-modifiable cardiovascular risk factors such as advanced age, male sex, as well as smoking and renal failure, were independently associated with CAD complexity assessed by SS. SS may be a valid marker of CAD extension to establish relationships with potential novel biomarkers of coronary atherosclerosis.
